# Completude e perfil clínico-epidemiológico das notificações de acidentes por animais peçonhentos: análise descritiva, Centro-Sul Fluminense, 2020-2023

**DOI:** 10.1590/S2237-96222026v35e20250541.pt

**Published:** 2026-11-09

**Authors:** Laila Fieto Ribeiro, Elder Damasceno Moura, Paloma Carvalho Guimarães, Thiago de Almeida Rosa

**Affiliations:** 1 Faculdade de Ciências Médicas de Três Rios, Três Rios, RJ, Brasil

**Keywords:** Animais Peçonhentos, Notificação Compulsória, Epidemiologia, Soro Antiveneno, Sistemas de Informação em Saúde, Animales Venenosos, Notificación de Enfermedades, Epidemiología, Antivenenos, Sistemas de Información en Salud

## Abstract

**Objetivos:**

Descrever o perfil epidemiológico de acidentes envolvendo animais peçonhentos e avaliar a completude das fichas de notificação e o uso da soroterapia conforme indicação em um polo de distribuição de soros do Centro-Sul Fluminense, entre 2020 e 2023.

**Métodos:**

Estudo transversal e descritivo, com análise das fichas de acidentes por animais peçonhentos registrados na Unidade de Pronto Atendimento de Três Rios, Rio de Janeiro. Todas as informações contidas nas fichas de notificação e a completude dos campos foram observadas. Calcularam-se frequências absolutas e relativas para variáveis categóricas e medidas de tendência central e de dispersão para variáveis numéricas.

**Resultados:**

Foram analisadas 293 fichas. Escorpiões foram responsáveis por 68,9% dos acidentes, seguidos por aranhas (14,0%) e serpentes (13,7%). A maioria dos casos envolveu homens (50,5%) e pessoas com idade entre 13-39 anos (38,2%), e o atendimento ocorreu em até três horas (67,1%). As picadas ocorreram principalmente em mãos (escorpionismo: 47,0%) e pés (araneísmo: 34,2%; ofidismo: 40,0%). A maioria dos casos foi leve (ofidismo: 40,0%) ou apresentou classificação ignorada (escorpionismo: 36,1%; araneísmo: 51,2%). Houve administração de soro em casos leves de escorpionismo (26,9%) e araneísmo (36,4%). A completude dos campos complicações locais (90,8% de incompletude), complicações sistêmicas (73,7% de incompletude) e escolaridade (91,5% de incompletude) foi classificada como muito ruim.

**Conclusão:**

Observaram-se predominância de escorpionismo, elevada incompletude dos registros e uso de soroterapia em casos leves, prática não recomendada. Esses achados reforçaram a necessidade de aprimorar o preenchimento das fichas e capacitar as equipes, fortalecendo a vigilância e o uso racional dos soros antiveneno.

## Resumen

**Objetivos:**

Describir el perfil epidemiológico de los accidentes causados por animales ponzoñosos y evaluar la completitud de las fichas de notificación y el uso de la terapia con suero según la indicación en un polo de distribución de sueros del Centro-Sur del Estado de Río de Janeiro, entre 2020 y 2023.

**Métodos:**

Estudio transversal y descriptivo, con análisis de las fichas de accidentes por animales ponzoñosos registradas en la Unidad de Pronto Atención de Três Rios, Río de Janeiro. Se observaron todas las informaciones contenidas en las fichas de notificación y la completitud de los campos. Se calcularon frecuencias absolutas y relativas para variables categóricas y medidas de tendencia central y de dispersión para variables numéricas.

**Resultados:**

Se analizaron 293 fichas. Los escorpiones fueron responsables del 68,9% de los accidentes, seguidos por las arañas (14,0%) y las serpientes (13,7%). La mayoría de los casos involucró a hombres (50,5%) y a personas de entre 13 y 39 años (38,2%), y la atención se realizó en un plazo de hasta tres horas (67,1%). Las picaduras ocurrieron principalmente en las manos (alacranismo: 47,0%) y en los pies (araneísmo: 34,2%; ofidismo: 40,0%). La mayoría de los casos fue leve (ofidismo: 40,0%) o presentó una clasificación no especificada (alacranismo: 36,1%; araneísmo: 51,2%). Se administró suero en casos leves de alacranismo (26,9%) y de araneísmo (36,4%). La completitud de los campos de complicaciones locales (90,8% de incompletitud), de complicaciones sistémicas (73,7% de incompletitud) y de escolaridad (91,5% de incompletitud) fue clasificada como muy deficiente.

**Conclusión:**

Se observó predominio del alacranismo, elevada incompletitud de los registros y el uso de terapia con suero en casos leves, práctica no recomendada. Estos hallazgos refuerzan la necesidad de mejorar el llenado de las fichas y de capacitar a los equipos, a fin de fortalecer la vigilancia y el uso racional de los sueros antiveneno.

## Introdução

Os envenenamentos por animais peçonhentos constituem um considerável problema de saúde pública mundial, passando a ser reconhecido, em 2007, como um agravo negligenciado [1]. Estimou-se a ocorrência anual de 3,5 milhões de acidentes por animais peçonhentos em todo o mundo, resultando em aproximadamente 125 mil óbitos relacionados apenas aos acidentes ofídicos [1]. Os principais animais peçonhentos de interesse da saúde pública compreendem espécies de escorpiões, aranhas e serpentes [2]. Apenas no estado do Rio de Janeiro, entre 2019 e 2024, foram notificados 13.898 casos de acidentes com animais peçonhentos, o que resultou em 48 óbitos nesse período [2].

Esses agravos são monitorados pelo Sistema Único de Saúde, por meio do Sistema de Informação de Agravos de Notificação (Sinan), que utiliza ficha padronizada para coletar e sistematizar os dados. A ficha de notificação compulsória reúne dados pessoais e características do acidente, como o intervalo entre picada e atendimento, local do corpo acometido, zona de ocorrência, tipo de animal envolvido, soroterapia, complicações e manifestações clínicas [3]. Determinadas informações exigem preenchimento obrigatório, e a falta desses dados inviabiliza a inclusão ou o processamento da notificação no Sinan, enquanto outras são consideradas de preenchimento essencial, sendo importantes para investigar o caso ou para calcular indicadores epidemiológicos [3].

A logística para a distribuição dos soros antivenenos, produzidos em institutos nacionais, como o Instituto Butantan, a Fundação Ezequiel Dias e o Instituto Vital Brazil, baseia-se nas informações extraídas das notificações realizadas por meio do preenchimento dessas fichas. Isso permite a distribuição adequada pelo Ministério da Saúde, por intermédio das Secretarias Estaduais de Saúde [4]. É imprescindível a manutenção da base de dados do Sinan atualizada, considerando que a implementação de estratégias que envolvam disponibilidade dos soros antivenenos, percepção sobre a necessidade de capacitação dos profissionais de saúde e de promoção de campanhas preventivas dependem da qualidade dessas informações [5].

Compreender a dinâmica local dos acidentes por animais peçonhentos torna-se fundamental para subsidiar a tomada de decisão e o aprimoramento da vigilância epidemiológica. Três Rios, localizada na região Centro-Sul Fluminense, é uma das três cidades da região que atuam como polo de referência para a distribuição de soros antivenenos para cidades vizinhas [6]. O município possui 326,136 km² e, em 2022, possuía 78.346 habitantes [7]. Três Rios apresenta alguns pequenos trechos de vegetação natural, sendo expressivamente fragmentados devido ao rápido crescimento industrial e populacional na cidade, além da degradação decorrente da atividade agropecuária [8]. Devido a essa perda de habitat natural, muitos animais silvestres tendem a refugiarem-se nos centros urbanos [9,10]. Desse modo, espera-se o aumento na incidência de acidentes causados por animais peçonhentos no município. Entretanto, Três Rios carece de informações atualizadas e de estudos mais completos sobre esse tipo de acidente.

Embora o Ministério da Saúde estabeleça critérios específicos para usar soros antivenenos, a avaliação da aderência às recomendações de soroterapia e da adequação das doses aplicadas permanece relevante para analisar a assistência prestada às vítimas de acidentes por animais peçonhentos. A avaliação da completude das fichas de notificação, associada à análise dessas práticas, pode contribuir para identificar fragilidades na qualidade das informações e no manejo clínico dos casos, aspectos fundamentais para planejar a vigilância e racionalizar os recursos disponíveis. 

Este estudo teve como objetivos descrever o perfil epidemiológico dos acidentes causados por animais peçonhentos e avaliar a completude das fichas de notificação e a adequação da administração dos soros em um polo de distribuição da região Centro-Sul Fluminense, no período 2020-2023.

## Métodos

### Delineamento do estudo

Trata-se de estudo transversal e descritivo, desenvolvido a partir das informações contidas nas fichas de notificação de acidentes por animais peçonhentos registradas no polo de distribuição de soros localizado em Três Rios, no estado do Rio de Janeiro, entre janeiro de 2020 e agosto de 2023. A escolha desse período justificou-se pelo fato de ser o intervalo em que as fichas foram disponibilizadas pela Secretaria Municipal de Vigilância Epidemiológica de Três Rios para consulta dos pesquisadores. 

Este estudo observacional foi descrito de acordo com as recomendações do checklist Strengthening the Reporting of Observational Studies in Epidemiology (STROBE) [11].

### Contexto

O polo de distribuição de soros está sediado na Unidade de Pronto Atendimento de Três Rios, que atua como centro de referência para atender acidentes por animais peçonhentos na região Centro-Sul Fluminense. O serviço integra a rede de vigilância em saúde estadual e recebe usuários provenientes de diferentes municípios da região. Três Rios e cidades vizinhas, como Areal, Comendador Levy Gasparian e Paraíba do Sul, apresentam ocupação urbana concentrada e extensas áreas rurais próximas, com predominância de áreas de uso agrícola, pastagem e remanescentes florestais.

### Participantes

Foram incluídas todas as fichas de notificação emitidas entre 1º de janeiro de 2020 e 31 de agosto de 2023, correspondendo a um censo dos registros disponíveis no serviço. Foram excluídas fichas com informações divergentes do receituário apensado, evitando possíveis erros de registro.

### Variáveis

As variáveis analisadas incluíram: município de ocorrência do acidente, sexo, faixa etária, classificação do caso, tipo de acidente, realização de soroterapia, gravidade, manifestações clínicas, tipo de soro e quantidade de ampolas aplicadas.

### Fonte de dados

As informações foram obtidas das fichas de notificação compulsória, que são preenchidas por médicos ou enfermeiros, paralelamente ao atendimento do usuário na Unidade de Pronto Atendimento. Posteriormente, são encaminhadas ao Núcleo de Vigilância Epidemiológica municipal, setor responsável pela organização e conferência dos dados a partir dos prontuários dos usuários e pelo envio dos registros à Secretaria de Estado de Saúde. 

A extração dos dados foi realizada entre setembro de 2021 e setembro de 2023, diretamente no Núcleo de Vigilância Epidemiológica de Três Rios, por pesquisadores autorizados. Quando as fichas não continham o tipo de soro ou a quantidade de ampolas aplicadas, esses dados foram extraídos dos receituários apensados.

### Tamanho do estudo

Foram analisadas 293 fichas de notificação de acidentes por animais peçonhentos, preenchidas entre janeiro de 2020 e agosto de 2023.

### Métodos estatísticos

A completude dos registros foi avaliada segundo a classificação proposta por Romero e Cunha [12], que definiram categorias conforme a proporção de dados incompletos: excelente (≤5,0%), boa (5,0%-10,0%), regular (10,0%-20,0%), ruim (20,0%-50,0%) e muito ruim (>50,0%). Realizou-se análise descritiva das variáveis, com cálculo de frequências absolutas e relativas para as variáveis categóricas e de média e desvio-padrão para a variável “quantidade de ampolas”. 

Para apresentar a sazonalidade das notificações, a média mensal de acidentes foi obtida somando-se o total de notificações registradas em cada mês e dividindo-se esse total pelo número de meses incluídos na análise, considerando o período de janeiro de 2020 a agosto de 2023. Os dados foram digitados, organizados e analisados no Microsoft Excel 2010. O banco de dados está disponível no repositório Figshare [13].

## Resultados

Foram analisadas 293 fichas de notificação de acidentes por animais peçonhentos. Destas, 283 (96,6%) correspondiam a acidentes causados por escorpiões, aranhas ou serpentes. Houve 1 caso por lagarta e 1 por lacraia. O campo “tipo de animal” não foi preenchido em 2,7% das fichas. Predominaram os casos por escorpiões (68,9%), seguidos por aranhas (14,0%) e serpentes (13,7%) (Tabela 1). Nenhum óbito foi registrado no período analisado.

Observou-se predominância do sexo masculino no conjunto dos acidentes (50,5%), com destaque para os acidentes ofídicos, nos quais 72,5% ocorreram em homens e 22,5% em mulheres; 5,0% das fichas não continham essa informação (Tabela 1). A maior incidência de escorpionismo foi em outubro (11,7 casos, em média), de araneísmo em fevereiro e novembro (2,0 casos, em média) e de ofidismo em janeiro (2,7 casos em média) (Figura 1).

**Tabela 1:** Distribuição dos acidentes com animais peçonhentos (escorpião, aranha e serpente), segundo variáveis sociodemográficas e clínicas. Três Rios, 2020-2023 (n=283)

Tipo de animal	Escorpião	Aranha	Serpente	Total
	n (%)	n (%)	n (%)	n (%)
Total de casos	202 (68,9)	41 (14,0)	40 (13,7)	283 (100)
Faixa etária (anos)				
<12	25 (12,4)	6 (14,6)	3 (7,5)	34 (12,0)
13-39	77 (38,1)	15 (36,6)	16 (40,0)	108 (38,2)
40-59	46 (22,8)	12 (29,3)	13 (32,5)	71 (25,1)
>60	31 (15,3)	6 (14,6)	7 (17,5)	44 (15,5)
Ignorado/em branco	23 (11,4)	2 (4,9)	1 (2,5)	26 (9,2)
Sexo				
Masculino	91 (45,1)	23 (56,1)	29 (72,5)	143 (50,5)
Feminino	81 (40,1)	17 (41,5)	9 (22,5)	107 (37,8)
Ignorado/em branco	30 (14,8)	1 (2,4)	2 (5,0)	33 (11,7)
Acidente de trabalho				
Sim	9 (4,5)	1 (2,4)	3 (7,5)	13 (4,6)
Não	38 (18,8)	7 (17,1)	8 (20,0)	53 (18,7)
Ignorado/em branco	155 (76,7)	33 (80,5)	29 (72,5)	217 (76,7)
Zona de ocorrência				
Urbana	95 (47,0)	15 (36,6)	7 (17,5)	117 (41,3)
Rural	71 (35,2)	19 (46,3)	27 (67,5)	117 (41,3)
Ignorado/em branco	36 (17,8)	7 (17,1)	6 (15,0)	49 (17,3)
Local da picada				
Pé/dedo do pé	39 (19,3)	14 (34,2)	16 (40,0)	69 (24,4)
Perna/coxa	20 (9,9)	6 (14,6)	7 (17,5)	33 (11,7)
Mão/dedo da mão	95 (47,0)	11 (26,8)	10 (25,0)	116 (41,0)
Braço/antibraço/axila	20 (9,9)	2 (4,9)	4 (10,0)	26 (9,2)
Outros	20 (9,9)	3 (7,3)	0 (0,0)	23 (8,1)
Ignorado/em branco	8 (4,0)	5 (12,2)	3 (7,5)	16 (5,7)
Tempo da picada até assistência (horas)				
0-1	82 (40,6)	9 (22,0)	5 (12,5)	96 (33,9)
1-3	60 (29,7)	13 (31,7)	21 (52,5)	94 (33,2)
3-6	15 (7,4)	5 (12,2)	4 (10,0)	24 (8,5)
6-12	5 (2,5)	0 (0,0)	1 (2,5)	6 (2,1)
>12	0 (0,0)	6 (14,6)	2 (5,0)	8 (2,8)
Ignorado/em branco	40 (19,8)	8 (19,5)	7 (17,5)	55 (19,4)
Severidade				
Leve	67 (33,2)	11 (26,8)	16 (40,0)	94 (33,2)
Moderado	55 (27,2)	9 (22,0)	9 (22,5)	73 (25,8)
Grave	7 (3,5)	0 (0,0)	5 (12,5)	12 (4,2)
Ignorado/em branco	73 (36,1)	21 (51,2)	10 (25,0)	104 (36,7)
Sinais e sintomas locais				
Dor	179 (88,6)	31 (75,6)	28 (70,0)	238 (84,1)
Edema	83 (41,1)	20 (48,8)	23 (55,0)	126 (44,5)
Parestesia	7 (3,5)	3 (7,3)	3 (7,5)	13 (4,6)
Eritema/hiperemia/rubor	6 (3,0)	0 (0,0)	0 (2,5)	6 (2,1)
Sinais e sintomas sistêmicos				
Anúria, oligúria, colúria	2 (1,0)	0 (0,0)	1 (2,5)	3 (1,1)
Febre	1 (0,5)	1 (2,4)	0 (0,0)	2 (0,7)
Hemorragias	0 (0,0)	0 (0,0)	2 (5,0)	2 (0,7)
Hipertensão	2 (1,0)	2 (4,9)	0 (0,0)	4 (1,4)
Mialgia	2 (1,0)	0 (0,0)	1 (2,5)	3 (1,1)
Ptose palpebral	1 (0,5)	0 (0,0)	2 (5,5)	3 (1,1)
Sintomas vagais (vômito, diarreia)	12 (5,4)	2 (4,9)	0 (0,0)	14 (4,9)
Sudorese	5 (2,5)	1(2,4)	0 (0,0)	6 (2,1)
Taquicardia	4 (2,0)	1 (2,4)	1 (2,5)	6 (2,1)
Taquipneia	1 (0,5)	0 (0,0)	0 (0,0)	1 (0,4)
Turvação visual	1 (0,5)	0 (0,0)	2 (5,0)	3 (1,1)
Ignorado/em branco	176 (87,1)	35 (85,4)	32 (80,0)	243 (85,9)

**Figura 1:**
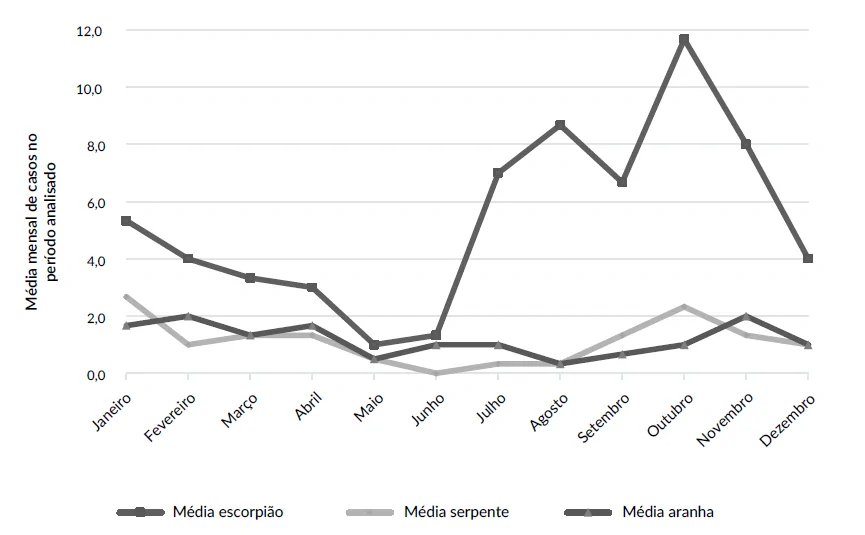
Sazonalidade dos acidentes por tipo de animal durante os quatro anos investigados, considerando a média mensal de casos. Três Rios, 2020-2023 (n=283)

A maior incidência de acidentes envolvendo escorpião ocorreu em indivíduos do sexo masculino (45,1%) e em adultos jovens (13-39 anos; 38,1%). As picadas ocorreram principalmente nas mãos/dedos das mãos (47,0%), e 40,6% das vítimas procuraram atendimento médico na primeira hora após a picada (Tabela 1).

Em relação à gravidade, prevaleceram as formas leves (33,2%) e moderadas (27,2%). Uma quantidade relevante de fichas não apresentava o campo “gravidade” preenchido (36,1%). Dor (88,6%) e edema (41,1%) foram as manifestações locais mais frequentes. As manifestações sistêmicas mais evidenciadas foram sintomas vagais como vômito e diarreia (n=12; 5,4%), seguido de sudorese (2,5%) e taquicardia (2,0%) (Tabela 1).

Nos acidentes por aranhas, verificou-se predominância masculina (56,1%) e da faixa etária de 13-39 anos (36,6%). Os locais da picada mais frequentes foram pé/dedos do pé (34,2%) e mão/dedos da mão (26,8%), e o atendimento ocorreu mais frequentemente entre uma e três horas após o envenenamento (31,7%). A maioria das fichas de notificação não apresentava o campo “gravidade do caso” preenchido (51,2%). Predominaram os casos classificados como leves (26,8%) e moderados (22,0%), sem registros de caso grave. Os sinais e os sintomas locais mais observados foram dor (75,6%), seguido de edema (48,8%) e, entre os sistêmicos, hipertensão, vômito e diarreia (4,9%) (Tabela 1). A maioria dos casos foram classificados como leves (40,0%) (Tabela 1).

Nos acidentes ofídicos, prevaleceram homens (72,5%) de 13-39 anos (40,0%), com picadas principalmente no pé/dedos do pé (40,0%), e 52,5% buscaram atendimento entre 1 e 3 horas após o acidente. Quarenta por cento foram classificados como leves, seguidos de 22,5% como moderados e 12,5% como graves; em 25,0% dos registros, a gravidade não foi informada. Dor (70,0%) e edema (55,0%) foram as manifestações locais mais frequentes. Entre os sintomas sistêmicos, observaram-se ptose palpebral e turvação visual em 2 casos (1 crotálico e 1 não identificado), além de taquicardia, mialgia e hemorragias gengivais em ocorrências isoladas (Tabela 1).

O tipo de aranha mais frequente foi a aranha-marrom (*Loxosceles* spp.) (n=7; 17,1%), associada ao loxoscelismo, seguida da aranha-armadeira (*Phoneutria* spp.) (n=4; 9,8%), associada ao foneutrismo, embora o campo “tipo de aranha” estivesse ausente em 58,5% das fichas. No caso dos acidentes ofídicos, jararacas foram as serpentes responsáveis por 40,0% dos casos, seguidas pelas cascáveis (30,0%). Houve um registro de usuário acometido por cobra-coral, e a espécie não foi informada em 27,5% das notificações (Tabela 2).

**Tabela 2:** Frequência absoluta e relativa dos casos registrados de araneísmo (especificado pelo tipo de aranha) e ofidismo (especificado pelo tipo de serpente). Três Rios, 2020-2023

Araneísmo - tipo de acidente (n=41)	n (%)
Loxoscelismo^a^	7 (17,1)
Foneutrismo^b^	4 (9,8)
Outra aranha	6 (14,6)
Ignorado/em branco	24 (58,5)
Ofidismo - tipo de acidente (n=40)	n (%)
Botrópico^c^	16 (40,0)
Crotálico^d^	12 (30,0)
Elapídico^e^	1 (2,5)
Ignorado/em branco	11 (27,5)

a Loxoscelismo: acidentes causados por aranhas-marrons (gênero *Loxosceles*); ^b^Foneutrismo: acidentes causados por aranhas-armadeiras (gênero *Phoneutria*); ^c^Botrópico: acidentes causados por jararacas (gênero *Bothrops*); ^d^Crotálico: acidentes causados por cascavéis (gênero *Crotalus*); ^e^Elapídico: acidentes causados por corais-verdadeiras (gênero *Micrurus*).

[Table t3]

**Tabela 3 t3:** Tipo de soro administrado nos acidentes com animais peçonhentos, segundo classificação do caso, quantidade de ampolas administradas e recomendações do Ministério da Saúde. Três Rios, 2020-2023 (n=283)

Soroterapia	Total de ampolas	Classificação do caso	Total de usuários	Média de ampolas (desvio-padrão)	Quantidade de ampolas recomendadas
Antiescorpiônico	251	Leve	18	2,2 (0,4)	-
		Moderado	50	2,2 (0,6)	2-3
		Grave	7	4,9 (1,1)	4-6
		Ignorado	29	2,4 (0,9)	-
Antiaracnídico	79	Leve	3	3,6 (1,7)	-
		Moderado	11	3,4 (1,2)	2-4
		Grave	-	-	5-10
		Ignorado	9	3,8 (1,1)	-
Antiloxoscélico	12	Leve	1	5	-
		Moderado	1	5	5
		Grave	-	-	10
		Ignorado	1	2	-
Antibotrópico	92	Leve	10	3,2 (0,9)	2-4
		Moderado	3	3,3 (2,3)	4-8
		Grave	2	3,3 (2,6)	12
		Ignorado	9	3,3 (1,1)	-
Anticrotálico	88	Leve	5	5,0 (0,0)	5
		Moderado	5	8,6 (4,7)	10
		Grave	1	20	20
		Ignorado	-	-	-
Antibotrópico - crotálico	20	Leve	1	10	2-4
		Moderado	1	10	4-8
		Grave	-	-	12
		Ignorado	-	-	-
Antielapídico	10	Leve	-	-	-
		Moderado	-	-	-
		Grave	1	10	10
		Ignorado	-	-	-

**Tabela 4:** Distribuição do percentual e escore de completude dos campos de preenchimento obrigatórios e essenciais da ficha de notificação de acidentes com animais peçonhentos. Três Rios, 2020-2023 (n=283)

Campo de preenchimento obrigatório	%	Escore
Nome completo	0,0	Excelente
Data de nascimento	4,8	Excelente
Idade	8,9	Boa
Sexo	11,3	Regular
Unidade da Federação	2,4	Excelente
Município	2,0	Excelente
Data do acidente	23,5	Ruim
Local da picada	6,5	Boa
Manifestações locais	10,6	Regular
Manifestações sistêmicas	85,3	Muito ruim
Tipo de acidente	2,7	Excelente
Serpente - tipo acidente	27,5	Ruim
Aranha - tipo acidente	58,5	Muito ruim
Tipo de soroterapia	42,7	Ruim
Complicações locais	90,8	Muito ruim
Complicações sistêmicas	73,7	Muito ruim
Data do encerramento	97,6	Muito ruim
Campo de preenchimento essencial		
Raça/cor	48,8	Ruim
Escolaridade	91,5	Muito ruim
Ocupação	55,3	Muito ruim
Zona de ocorrência	18,8	Regular
Tempo decorrido picada/atendimento	19,8	Regular
Classificação do caso	37,2	Ruim
Evolução do caso	95,6	Muito ruim

A análise da completude das fichas de notificação evidenciou elevada proporção de campos incompletos ou ignorados. Em relação aos campos obrigatórios, manifestações sistêmicas (85,3%), complicações locais (90,8%) e sistêmicas (73,7%) e data do encerramento (97,6%) apresentaram as maiores taxas de incompletude, sendo classificados como “muito ruins”. Os tipos de aranha e de serpente envolvidos nos casos também apresentaram incompletude elevada (58,5% e 25,5%). Já se tratando dos campos de preenchimento essencial, evolução do caso (95,6%) e escolaridade (91,5%) se destacaram como os campos mais incompletos, sendo classificados como “muito ruins”. Raça/cor (48,8%) e classificação do caso (37,2%) apresentaram preenchimento “ruim” (Tabela 4).

## Discussão

Este trabalho contribuiu para entender a epidemiologia dos acidentes por animais peçonhentos na região Centro-Sul Fluminense, revelando a predominância do escorpionismo, maior acometimento em homens, adultos jovens e lesões em extremidades. Foram identificadas duas fragilidades no manejo desses agravos: a aplicação de soroterapia em casos sem indicação e a elevada incompletude das fichas de notificação, ambas com implicações diretas para a vigilância, o uso racional de recursos e a segurança do usuário. Isso reforçou a necessidade de aprimorar as práticas assistenciais e o registro epidemiológico.

A maior incidência de escorpionismo, seguido por aranhas e serpentes, representou padrão similar ao observado em outras regiões do Brasil [14]. Escorpiões apresentaram grande capacidade de adaptação a ambientes antropizados e alta taxa reprodutiva, o que aumentou a incidência de envenenamentos em cidades com ocupação urbana concentrada [15]. Considerando todos os acidentes, a faixa etária predominante foi de pessoas entre 13-39 anos, semelhante a outros levantamentos epidemiológicos brasileiros [15,16]. Esse padrão é justificado pela maior mobilidade e pelo envolvimento em atividades de risco, como agricultura e construção civil, que expõem mais esses indivíduos [15-17]. Porém, a falta de preenchimento do campo sobre vínculo com o trabalho em 217 fichas limitou a análise dessa relação.

O levantamento indicou que a mão/dedos das mãos foi a região mais afetada por escorpionismo, enquanto pés/dedos dos pés foram as áreas mais atingidas por araneísmo e ofidismo. Esses achados foram compatíveis com o comportamento dos animais, pois escorpiões frequentemente se abrigam em entulhos e frestas, o que aumenta a chance de picadas durante a manipulação de objetos, enquanto aranhas e serpentes tendem a se localizar próximas ao solo, inclusive dentro de calçados [18]. Em áreas periurbanas e rurais, o costume de andar descalço possivelmente elevou o risco de acidentes por aranhas e serpentes. Entre essas serpentes, destacaram-se as espécies dos gêneros *Bothrops* e *Crotalus*, de hábito terrestre e críptico, predispondo a acidentes em membros inferiores quando são pisadas [18]. Esse padrão é relevante para orientar ações educativas, como uso de calçados, inspeção de sapatos e manejo adequado de resíduos.

Os resultados demonstraram que a maioria das vítimas procurou tratamento em até três horas após a picada, com grande parte dos casos classificados como leves, independentemente do tipo de animal peçonhento. Tal achado foi positivo, pois a eficácia do tratamento depende da busca rápida por atendimento, sendo essencial para um prognóstico favorável [19]. Um ponto preocupante foi a incompletude do campo “gravidade do acidente”, já que grande parte das fichas permaneceu em branco, o que dificultou a confirmação se a maioria dos casos foi realmente leve, especialmente nos acidentes envolvendo escorpiões e aranhas. 

A falta de informação sobre a espécie de aranha foi uma das principais deficiências identificadas, pois a maioria das fichas apresentou esse campo em branco ou ignorado, padrão já encontrado [17]. Isso reforça a necessidade de treinamento para o reconhecimento das manifestações clínicas relacionadas ao tipo de animal e, quando possível, identificar o animal diretamente ou por fotos trazidas pelos usuários. Entre os casos que tiveram identificação, *Loxosceles* foi o gênero que mais causou acidentes, como já observado [15,17]. A predominância desse gênero tem importância clínica significativa, pois o envenenamento por aranha-marrom esteve associado a quadros potencialmente graves, com risco de complicações sistêmicas e óbito [17]. 

Entre as serpentes, os acidentes com jararacas foram mais frequentes (40%), devido à ampla distribuição e ao comportamento agressivo do gênero *Bothrops* [18]. Embora as jararacas sejam responsáveis pela maioria dos envenenamentos ofídicos no Brasil, correspondendo a cerca de 70% dos casos [20], observou-se o aumento de acidentes crotálicos no interior do estado do Rio de Janeiro, que passaram de 9% para 40% dos casos ofídicos ao longo de sete anos de investigação [21]. No período investigado, as ocorrências com jararacas (40%) e cascavéis (30%) foram semelhantes. Cascavéis foram as serpentes que representaram maior risco de óbito na região Sudeste [2] devido ao efeito neurotóxico do veneno. Esse resultado acende um alerta para a necessidade da alta disponibilidade de soro anticrotálico, bem como para o fortalecimento da rede de atendimento capaz de reconhecer precocemente os sintomas de maior gravidade nesse tipo de acidente.

Observou-se maior ocorrência de escorpionismo no final da estação seca e em meses quentes [16,17]. Entretanto, não foi encontrada nítida flutuação na incidência de casos que envolveram aranhas e serpentes nas diferentes estações do ano, como pode ocorrer em regiões onde a sazonalidade climática é mais acentuada, como regiões amazônicas ou semiáridas [15,22]. A variação temporal dos acidentes não foi uniforme em todo o território nacional; em áreas de ocupação territorial relativamente estável e atividade humana contínua, o risco de acidentes pode se distribuir ao longo de todos os meses [15,17]. A baixa variabilidade na média de araneísmo e ofidismo e a constância desses tipos de envenenamento durante o ano devem ser consideradas ao planejar a disponibilidade de soros para tratamento durante todas as estações. 

Destacou-se a administração de soro antiveneno em casos leves de escorpionismo (n=18; 26,9%) e araneísmo (n=4; 36,4%). Isso contrariou diretrizes que indicam esse uso apenas para quadros moderados e graves nesses tipos de agravos [17]. Esse resultado revelou discrepância importante entre as recomendações técnicas e a prática assistencial observada no serviço analisado. A administração de soro antiveneno em casos sem indicação expõe o usuário a riscos relevantes, como reações anafiláticas e doença do soro, além de impactar negativamente tanto pelo desperdício desse recurso quanto pelo risco de comprometer a disponibilidade de estoques para casos realmente necessários [22]. 

O uso indevido de soroterapia ainda é frequente nos serviços de urgência e emergência, especialmente quando há insegurança diagnóstica ou desconhecimento das recomendações clínicas [22,24]. Essa constatação aponta para a necessidade de investimento contínuo na capacitação das equipes de saúde, com ênfase nas diretrizes de manejo clínico, classificação de gravidade e indicação precisa de soroterapia.

A elevada incompletude observada nas fichas de notificação, especialmente nos campos manifestações sistêmicas, complicações locais, complicações sistêmicas, escolaridade, raça/cor, ocupação e evolução do caso, classificados como “ruins” ou “muito ruins”, representou outro achado importante deste estudo. O preenchimento insatisfatório de campos ligados a determinantes sociais, como escolaridade, ocupação e raça/cor, dificulta a formulação de políticas públicas relacionadas à implementação de estratégias de prevenção desses acidentes voltadas à população mais vulnerável [24,25]. A ausência de informação sobre a evolução dos casos e sobre a ocorrência de complicações dificulta a mensuração da gravidade dos acidentes e, por conseguinte, pode comprometer o planejamento e a distribuição adequada dos soros antivenenos [24].

No caso dos campos de preenchimento obrigatório, a elevada proporção de registros em branco nas fichas físicas sugere fragilidades no processo de registro das informações. Considerando que esses campos são necessários para incluir e encerrar os casos no Sinan [4], o cenário encontrado indica a necessidade de maior atenção à etapa de registro e à supervisão desse processo [26]. Uma hipótese é que, em campos clínicos, a ausência de sinais e sintomas leve os profissionais a deixarem o campo em branco em vez de marcar “não”.

A falta de compreensão sobre a importância dos registros e a ausência de supervisão contribuíram para a baixa completude [25,26]. A incompletude também reflete fragilidades de gestão, como a falta de mecanismos de monitoramento sistemático e de definição clara de responsabilidades sobre o preenchimento das fichas de notificação [24,27]. Diante desse contexto, é essencial capacitar as equipes de saúde para aprimorar o preenchimento das fichas e o manejo clínico dos casos. A educação permanente e auditorias periódicas podem melhorar a qualidade dos dados, padronizar informações registradas e reforçar o cumprimento das rotinas de vigilância [24-25,27]. A implementação da obrigatoriedade ao preencher os campos considerados essenciais no Sinan reduziria substancialmente a incompletude das notificações e elevaria a confiabilidade dos indicadores gerados [24,25]. 

Entendeu-se como limitação deste estudo o uso de dados do período da pandemia de covid-19, marcado por isolamento social e menor busca por serviços de saúde não essenciais [28], o que pode ter causado subnotificação de acidentes por animais peçonhentos. Contudo, enquanto na região Sudeste foi identificada tendência estável na incidência desses agravos durante a pandemia [29], observou-se redução nos casos de escorpionismo e araneísmo no país [30], sugerindo que o impacto da pandemia sobre os registros pode ter variado regionalmente. As informações utilizadas neste estudo foram obtidas diretamente das fichas físicas de notificação, ainda não inseridas no Sinan, preenchidas por diferentes profissionais durante o atendimento. Portanto, o estudo esteve sujeito a limitações por utilizar dados secundários, como possíveis inconsistências e falhas no preenchimento dos campos investigados. 

Este estudo evidenciou a predominância do escorpionismo e a relevância desse agravo como problema persistente de saúde pública na região Centro-Sul Fluminense. Embora a maioria dos casos tenha sido classificada como leve e com rápido atendimento, observou-se a utilização de soroterapia em situações sem indicação clínica, o que evidenciou fragilidades no manejo terapêutico e na aplicação das diretrizes do Ministério da Saúde. Paralelamente, a elevada incompletude das fichas de notificação comprometeu a avaliação da gravidade dos acidentes e o planejamento da distribuição de soros. A ausência de identificação das espécies envolvidas reforça, ainda, a necessidade de capacitação técnica dos profissionais de saúde, visando melhorar o diagnóstico e o manejo terapêutico nos casos de envenenamento. Assim, aprimorar os registros e as condutas clínicas, com protocolos atualizados, treinamentos e supervisão frequentes, é fundamental para promover o uso racional dos recursos, fortalecer a vigilância e garantir atendimento mais seguro e eficiente aos usuários.

## Data Availability

O banco de dados utilizado na pesquisa está disponível no repositório Figshare através do link: https://doi.org/10.6084/m9.figshare.28655264 [12].
