# Supplementary material for: Completude e perfil clínico-epidemiológico das notificações de acidentes por animais peçonhentos: análise descritiva, Centro-Sul Fluminense, 2020-2023
Source: Epidemiol Serv Saude. 2026 Nov 9;35:e20250541. [Article in Portuguese] doi: 10.1590/S2237-96222026v35e20250541.pt (PMC13750718; doi:10.1590/S2237-96222026v35e20250541.pt)
Supplement: Translation

# Translation: Completeness and clinical-epidemiological profile of notifications of venomous animal envenomations: a descriptive analysis in the Centro-Sul region of the State of Rio de Janeiro, 2020-2023

**DOI:** 10.1590/S2237-96222026v35e20250541.en

**Authors:** Laila Fieto Ribeiro, Elder Damasceno Moura, Paloma Carvalho Guimarães, Thiago de Almeida Rosa

**Affiliations:** 1 Faculdade de Ciências Médicas de Três Rios, Três Rios, RJ, Brazil

**Keywords:** Venomous Animals, Disease Notification, Epidemiology, Antivenins, Health Information Systems

## Abstract

**Objectives:**

To describe the epidemiological profile of cases involving venomous animals and to assess the completeness of notification forms and the use of antivenom therapy according to indication in an antivenom distribution center in the Centro-Sul (Central-South) region of Rio de Janeiro State, between 2020 and 2023.

**Methods:**

A cross-sectional and descriptive study was conducted, with analysis of records of cases involving venomous animals registered at the Emergency Care Unit of Três Rios, Rio de Janeiro. All information contained in the notification forms and the completeness of the fields were assessed. Absolute and relative frequencies were calculated for categorical variables, and measures of central tendency and dispersion were calculated for numerical variables.

**Results:**

A total of 293 records were analyzed. Scorpions accounted for 68.9% of the cases, followed by spiders (14.0%) and snakes (13.7%). Most cases involved men (50.5%) and individuals aged 13-39 years (38.2%), and care was provided within up to three hours (67.1%). Bites occurred mainly on the hands (scorpionism: 47.0%) and feet (arachnidism: 34.2%; ophidism: 40.0%). Most cases were mild (ophidism: 40.0%) or had an unknown classification (scorpionism: 36.1%; arachnidism: 51.2%). Antivenom administration occurred in mild cases of scorpionism (26.9%) and arachnidism (36.4%). Field completeness was classified as very poor for local complications (90.8% incompleteness), systemic complications (73.7% incompleteness), and educational level (91.5% incompleteness).

**Conclusion:**

A predominance of scorpionism, high incompleteness of records, and the use of antivenom therapy in mild cases, a practice that is not recommended, were observed. These findings reinforce the need to improve form completion and to train healthcare teams, strengthening surveillance and the rational use of antivenom.

## Introduction

Envenomation caused by venomous animals constitutes a major global public health problem and was recognized in 2007 as a neglected health condition [1]. An estimated 3.5 million envenomations caused by venomous animals occur annually worldwide, resulting in approximately 125,000 deaths related solely to snakebite cases [1]. The main venomous animals of public health importance include species of scorpions, spiders, and snakes [2]. In the state of Rio de Janeiro alone, between 2019 and 2024, 13,898 envenomations caused by venomous animals were reported, resulting in 48 deaths during this period [2].

These conditions are monitored by the Brazilian Unified Health System (*Sistema Único de Saúde*, SUS), through the Notifiable Diseases Information System (*Sistema de Informação de Agravos de Notificação*, Sinan), which uses a standardized form to collect and systematize data. The compulsory notification form includes personal data and accident characteristics, such as the interval between the bite and care, the affected body site, the place of occurrence, the type of animal involved, antivenom therapy, complications, and clinical manifestations [3]. Certain fields are mandatory, and missing data prevents inclusion or processing of the notification in Sinan; other fields are considered essential because they are important for case investigation or for calculating epidemiological indicators [3].

The logistics for distributing antivenom sera produced at national institutes such as the Butantan Institute, the Ezequiel Dias Foundation (*Fundação Ezequiel Dias*, Funed), and the Vital Brazil Institute (*Instituto Vital Brazil*) are based on information extracted from notifications completed using these forms. This allows appropriate distribution by the Ministry of Health, through the State Health Departments [4]. Maintaining an up-to-date Sinan database is essential, as the implementation of strategies involving antivenom availability, awareness of the need for healthcare professional training, and the promotion of preventive campaigns depends on the quality of this information [5].

Understanding the local dynamics of envenomations caused by venomous animals is essential for decision-making and improving epidemiological surveillance. Três Rios, located in the Centro-Sul (Central-South) region of the State of Rio de Janeiro, is one of the three municipalities in the region that serve as reference centers for distributing antivenom to neighboring cities [6]. The municipality covers 326.136 km² and had a population of 78,346 inhabitants in 2022 [7]. Três Rios contains small remnants of natural vegetation, which are highly fragmented due to rapid industrial and population growth in the city, as well as environmental degradation resulting from agricultural activities [8]. Due to this loss of natural habitat, many wild animals tend to seek refuge in urban centers [9,10]. As a result, an increase in the incidence of cases caused by venomous animals in the municipality is expected. However, Três Rios lacks updated information and more comprehensive studies on this type of accident.

Although the Ministry of Health establishes specific criteria for antivenom use, assessing adherence to antivenom therapy recommendations and the appropriateness of administered doses remain relevant for evaluating the care provided to victims of envenomation by venomous animals. The assessment of notification form completeness, combined with the analysis of these practices, may help identify weaknesses in data quality and case management, both of which are essential for planning surveillance and optimizing available resources. 

This study aimed to describe the epidemiological profile of envenomations caused by venomous animals and to assess the completeness of notification forms and the adequacy of antivenom administration in a distribution hub in the Centro-Sul region of the State of Rio de Janeiro, from 2020 to 2023.

## Methods

### Study design

This is a cross-sectional, descriptive study based on information from notification forms for envenomations caused by venomous animals registered at the antivenom distribution center in Três Rios, state of Rio de Janeiro, between January 2020 and August 2023. The choice of this period was justified because it corresponds to the interval during which the forms were made available by the Municipal Epidemiological Surveillance Department of Três Rios for researchers’ consultation. 

This observational study was reported in accordance with the Strengthening the Reporting of Observational Studies in Epidemiology (STROBE) checklist [11].

### Setting

The antivenom distribution center is based at the Emergency Care Unit of Três Rios, which serves as a reference center for managing envenomations caused by venomous animals in the Centro-Sul region of the State of Rio de Janeiro. The service is part of the state health surveillance network and receives users from different municipalities in the region. Três Rios and neighboring municipalities, such as Areal, Comendador Levy Gasparian, and Paraíba do Sul, present concentrated urban occupation and extensive nearby rural areas, with a predominance of agricultural land use, pasture, and forest remnants.

### Participants

All notification forms issued between 1 January 2020 and 31 August 2023 were included, corresponding to a census of the records available in the service. Forms with information inconsistent with the attached prescription were excluded to avoid potential recording errors.

### Variables

The variables analyzed included: municipality where the accident occurred, sex, age group, case classification, type of accident, administration of antivenom therapy, severity, clinical manifestations, type of antivenom, and number of vials administered.

### Data sources

The information was obtained from compulsory notification forms completed by physicians or nurses concurrently with patient care in the Emergency Care Unit. Subsequently, they are forwarded to the Municipal Epidemiological Surveillance Center, the sector responsible for organizing and verifying data from patient records and submitting reports to the State Health Department. 

Data extraction was conducted between September 2021 and September 2023 at the Epidemiological Surveillance Center of Três Rios by authorized researchers. When forms did not contain the type of antivenom or the number of vials administered, these data were retrieved from the attached prescriptions.

### Study size

A total of 293 notification forms for envenomation caused by venomous animals, completed between January 2020 and August 2023, were analyzed.

### Statistical methods

Record completeness was assessed according to the classification proposed by Romero and Cunha [12], who defined categories based on the proportion of missing data: excellent (≤5.0%), good (5.0%-10.0%), fair (10.0%-20.0%), poor (20.0%-50.0%), and very poor (>50.0%). A descriptive analysis of the variables was performed, with calculation of absolute and relative frequencies for categorical variables and mean and standard deviation for the variable “number of vials.” 

To describe the monthly distribution of notifications, the mean number of cases per month was calculated by summing the total notifications recorded each month and dividing this total by the number of months in the analysis, covering the period from January 2020 to August 2023. Data were entered, organized, and analyzed using Microsoft Excel 2010. The dataset is available in the Figshare repository [13]. 

## Results

A total of 293 notification forms for envenomation caused by venomous animals were analyzed. Of these, 283 (96.6%) were caused by scorpions, spiders, or snakes. There was 1 case involving a caterpillar and 1 involving a centipede. The “type of animal” field was not completed in 2.7% of the forms. Scorpion-related cases predominated (68.9%), followed by spider-related (14.0%) and snakebite-related cases (13.7%) (Table 1). No deaths were recorded during the study period.

A predominance of males was observed across all cases (50.5%), particularly in snakebite cases, where 72.5% were men and 22.5% were women; 5.0% of the forms did not contain this information (Table 1). The highest incidence of scorpionism was in October (mean of 11.7 cases), of araneism in February and November (mean of 2.0 cases), and of ophidism in January (mean of 2.7 cases) (Figure 1).

**Table 1:** Distribution of envenomation cases caused by venomous animals (scorpion, spider, and snake bites) by sociodemographic and clinical variables. Três Rios, 2020-2023 (n=283)

Type of animal	Scorpion	Spider	Snake	Total
	n (%)	n (%)	n (%)	n (%)
Total cases	202 (68,9)	41 (14,0)	40 (13,7)	283 (100)
Age group (years)				
<12	25 (12,4)	6 (14,6)	3 (7,5)	34 (12,0)
13-39	77 (38,1)	15 (36,6)	16 (40,0)	108 (38,2)
40-59	46 (22,8)	12 (29,3)	13 (32,5)	71 (25,1)
>60	31 (15,3)	6 (14,6)	7 (17,5)	44 (15,5)
Unknown/blank	23 (11,4)	2 (4,9)	1 (2,5)	26 (9,2)
Sex				
Male	91 (45,1)	23 (56,1)	29 (72,5)	143 (50,5)
Female	81 (40,1)	17 (41,5)	9 (22,5)	107 (37,8)
Unknown/blank	30 (14,8)	1 (2,4)	2 (5,0)	33 (11,7)
Work-related accident				
Yes	9 (4,5)	1 (2,4)	3 (7,5)	13 (4,6)
No	38 (18,8)	7 (17,1)	8 (20,0)	53 (18,7)
Unknown/blank	155 (76,7)	33 (80,5)	29 (72,5)	217 (76,7)
Place of residence				
Urban area	95 (47,0)	15 (36,6)	7 (17,5)	117 (41,3)
Rural area	71 (35,2)	19 (46,3)	27 (67,5)	117 (41,3)
Unknown/blank	36 (17,8)	7 (17,1)	6 (15,0)	49 (17,3)
Bite/sting site				
Foot/toe	39 (19,3)	14 (34,2)	16 (40,0)	69 (24,4)
Leg/thigh	20 (9,9)	6 (14,6)	7 (17,5)	33 (11,7)
Hand/finger	95 (47,0)	11 (26,8)	10 (25,0)	116 (41,0)
Arm/forearm/axillary region	20 (9,9)	2 (4,9)	4 (10,0)	26 (9,2)
Other	20 (9,9)	3 (7,3)	0 (0,0)	23 (8,1)
Unknown/blank	8 (4,0)	5 (12,2)	3 (7,5)	16 (5,7)
Time from bite/sting to care (hours)				
0-<1	82 (40,6)	9 (22,0)	5 (12,5)	96 (33,9)
1-3	60 (29,7)	13 (31,7)	21 (52,5)	94 (33,2)
3-6	15 (7,4)	5 (12,2)	4 (10,0)	24 (8,5)
6-12	5 (2,5)	0 (0,0)	1 (2,5)	6 (2,1)
>12	0 (0,0)	6 (14,6)	2 (5,0)	8 (2,8)
Unknown/blank	40 (19,8)	8 (19,5)	7 (17,5)	55 (19,4)
Severity				
Mild	67 (33,2)	11 (26,8)	16 (40,0)	94 (33,2)
Moderate	55 (27,2)	9 (22,0)	9 (22,5)	73 (25,8)
Severe	7 (3,5)	0 (0,0)	5 (12,5)	12 (4,2)
Unknown/blank	73 (36,1)	21 (51,2)	10 (25,0)	104 (36,7)
Local signs and symptoms				
Pain	179 (88,6)	31 (75,6)	28 (70,0)	238 (84,1)
Edema	83 (41,1)	20 (48,8)	23 (55,0)	126 (44,5)
Paresthesia	7 (3,5)	3 (7,3)	3 (7,5)	13 (4,6)
Erythema/hyperemia/flushing	6 (3,0)	0 (0,0)	0 (2,5)	6 (2,1)
Systemic signs and symptoms				
Anuria, oliguria, choluria	2 (1,0)	0 (0,0)	1 (2,5)	3 (1,1)
Fever	1 (0,5)	1 (2,4)	0 (0,0)	2 (0,7)
Haemorrhages	0 (0,0)	0 (0,0)	2 (5,0)	2 (0,7)
Hypertension	2 (1,0)	2 (4,9)	0 (0,0)	4 (1,4)
Myalgia	2 (1,0)	0 (0,0)	1 (2,5)	3 (1,1)
Ptosis	1 (0,5)	0 (0,0)	2 (5,5)	3 (1,1)
Autonomic symptoms (vomiting and diarrhea)	12 (5,4)	2 (4,9)	0 (0,0)	14 (4,9)
Sweating	5 (2,5)	1(2,4)	0 (0,0)	6 (2,1)
Tachycardia	4 (2,0)	1 (2,4)	1 (2,5)	6 (2,1)
Tachypnea	1 (0,5)	0 (0,0)	0 (0,0)	1 (0,4)
Blurred vision	1 (0,5)	0 (0,0)	2 (5,0)	3 (1,1)
Unknown/blank	176 (87,1)	35 (85,4)	32 (80,0)	243 (85,9)

**Figure 1:** Seasonality of accidents by animal type over the four-year study period, based on the mean monthly number of cases. Três Rios, 2020-2023 (n=283)

The highest incidence of scorpion stings occurred among males (45.1%) and young adults (aged 13-39 years; 38.1%). Stings occurred mainly on the hands/fingers (47.0%), and 40.6% of victims sought medical care within the first hour after the sting (Table 1).

Regarding severity, mild (33.2%) and moderate (27.2%) cases predominated. A substantial proportion of forms did not have the “severity” field completed (36.1%). Pain (88.6%) and edema (41.1%) were the most frequent local manifestations. The most common autonomic manifestations were vomiting and diarrhea (n=12; 5.4%), followed by sweating (2.5%) and tachycardia (2.0%) (Table 1).

Among spider envenomation, males (56.1%) and individuals aged 13-39 years (36.6%) predominated. The most frequent bite sites were the foot/toes (34.2%) and hand/fingers (26.8%), and care was most frequently provided between one and three hours after envenomation (31.7%). Most notification forms did not contain information in the “case severity” field (51.2%). Cases classified as mild (26.8%) and moderate (22.0%) predominated, with no severe cases recorded. The most frequently observed local signs and symptoms were pain (75.6%) and edema (48.8%); among systemic manifestations, hypertension, vomiting, and diarrhea predominated (4.9%) (Table 1). Most cases were classified as mild (40.0%) (Table 1).

Among snakebite envenomation cases, males (72.5%) and those aged 13-39 years (40.0%) predominated, bites occurred mainly on the foot/toes (40.0%), and 52.5% sought care between 1 and 3 hours after the accident. Forty percent were classified as mild, followed by 22.5% as moderate and 12.5% as severe; in 25.0% of records, severity was not reported. Pain (70.0%) and edema (55.0%) were the most frequent local manifestations. Among systemic manifestations, ptosis and blurred vision were observed in 2 cases (1 crotalic and 1 unidentified), with isolated occurrences of tachycardia, myalgia, and gingival bleeding (Table 1).

The most frequent spider type was the brown spider (*Loxosceles* spp.) (n=7; 17.1%), associated with loxoscelism, followed by the armed spider (*Phoneutria* spp.) (n=4; 9.8%), associated with phoneutrism, although the “type of spider” field was absent in 58.5% of the forms. Among snakebite accidents, lancehead snakes accounted for 40.0% of cases, followed by rattlesnakes (30.0%). There was one record of a user bitten by a coral snake, and the species was not reported in 27.5% of notifications (Table 2).

**Table 2:** Absolute and relative frequencies of recorded spider envenomation (by spider type) and snakebite envenomation (by snake type). Três Rios, 2020-2023

Arachnidism - type of envenomation (n=41)	n (%)
Loxoscelism^a^	7 (17,1)
Phoneutrism^b^	4 (9,8)
Another spider	6 (14,6)
Unknown/blank	24 (58,5)
Snakebite - type of envenomation (n=40)	n (%)
Botropic^c^	16 (40,0)
Crotalic^d^	12 (30,0)
Elapid^e^	1 (2,5)
Unknown/blank	11 (27,5)

a Loxoscelism: accidents caused by brown spiders (genus Loxosceles); ^b^Phoneutrism: accidents caused by wandering spiders (genus Phoneutria); ^c^Botropic: accidents caused by lancehead snakes (genus Bothrops); ^d^Crotalic: accidents caused by rattlesnakes (genus Crotalus); andElapid: accidents caused by coral snakes (genus Micrurus).

**Table 3:** Type of antivenom administered in envenomation cases caused by venomous animals, according to case classification, number of vials administered, and Ministry of Health recommendations. Três Rios, 2020-2023 (n=283)

Antivenom therapy	Total vials	Case classification	Total users	Average number of vials (standard deviation)	Recommended number of vials
Scorpion antivenom	251	Mild	18	2.2 (0.4)	-
		Moderate	50	2.2 (0.6)	2-3
		Severe	7	4.9 (1.1)	4-6
		Unknown	29	2.4 (0.9)	-
Arachnid antivenom	79	Mild	3	3.6 (1.7)	-
		Moderate	11	3.4 (1.2)	2-4
		Severe	-	-	5-10
		Unknown	9	3.8 (1.1)	-
Loxosceles antivenom	12	Mild	1	5	-
		Moderate	1	5	5
		Severe	-	-	10
		Unknown	1	2	-
Bothrops antivenom	92	Mild	10	3.2 (0.9)	2-4
		Moderate	3	3.3 (2.3)	4-8
		Severe	2	3.3 (2.6)	12
		Unknown	9	3.3 (1.1)	-
Crotalus antivenom	88	Mild	5	5.0 (0.0)	5
		Moderate	5	8.6 (4.7)	10
		Severe	1	20	20
		Unknown	-	-	-
Bothrops-Crotalus antivenom	20	Mild	1	10	2-4
		Moderate	1	10	4-8
		Severe	-	-	12
		Unknown	-	-	-
Micrurus antivenom	10	Mild	-	-	-
		Moderate	-	-	-
		Severe	1	10	10
		Unknown	-	-	-

**Table 4:** Distribution of the percentage and completeness score of mandatory and essential fields in notification forms for envenomation caused by venomous animals. Três Rios, 2020-2023 (n=283)

Mandatory field	%	Score
Full name	0.0	Excellent
Date of birth	4.8	Excellent
Age	8.9	Good
Sex	11.3	Fair
State and Federal District	2.4	Excellent
Municipality	2.0	Excellent
Date of the accident	23.5	Poor
Bite/sting site	6.5	Good
Local manifestations	10.6	Fair
Systemic manifestations	85.3	Very poor
Type of accident	2.7	Excellent
Snakebite - type of envenomation	27.5	Poor
Spider - type of envenomation	58.5	Very poor
Type of antivenom therapy	42.7	Poor
Local complications	90.8	Very poor
Systemic complications	73.7	Very poor
Case closure date	97.6	Very poor
Essential fields		
Race/color	48.8	Poor
Educational level	91.5	Very poor
Occupation	55.3	Very poor
Place of residence	18.8	Fair
Time from bite/sting to care	19.8	Fair
Case classification	37.2	Poor
Case outcome	95.6	Very poor

A total of 251 vials of scorpion antivenom were administered not only to severe and moderate cases but also to 18 of the 67 mild cases. Spider antivenom was also administered to 3 individuals with mild accidents and to 9 for whom severity information was unavailable. Twelve vials of Loxosceles antivenom (specific for brown spider envenomation) were administered, equally distributed between 1 individual with a mild case and 1 with a moderate case (both received 5 vials), while the remaining 2 vials were administered to 1 individual with no record of severity. For the treatment of snakebite envenomations, bothropic antivenom was the most frequently used (92 vials), followed by crotalic antivenom (88 vials). Elapid antivenom was administered to 91 individuals whose accident was classified as severe (Table 3).

Analysis of the notification form completeness revealed a high proportion of incomplete or missing fields. Among mandatory fields, systemic manifestations (85.3%), local complications (90.8%), systemic complications (73.7%), and case closure date (97.6%) presented the highest rates of incompleteness and were classified as “very poor.” The fields identifying the spider and snake species involved in the cases also showed high incompleteness (58.5% and 25.5%, respectively). Among essential fields, case outcome (95.6%) and educational level (91.5%) were the most incomplete and were classified as “very poor.” Race/color (48.8%) and case classification (37.2%) were classified as “poor” (Table 4).

## Discussion

This study contributed to understanding the epidemiology of envenomations caused by venomous animals in the Centro-Sul region of the State of Rio de Janeiro, revealing a predominance of scorpion stings, a higher incidence among males and young adults, and injuries affecting the extremities. Two weaknesses in the management of these health conditions were identified: the administration of antivenom therapy when it was not indicated and the high rate of incomplete notification forms, both of which have direct implications for surveillance, the rational use of resources, and patient safety. These findings reinforce the need to improve healthcare practices and epidemiological reporting.

The higher incidence of scorpion stings, followed by spider and snakebite cases, was similar to patterns observed in other regions of Brazil [14]. Scorpions show a high capacity to adapt to anthropized environments and have high reproductive rates, which increase the incidence of envenomations in cities with concentrated urban occupation [15]. Considering all accidents, the predominant age group was 13-39 years, similar to findings reported in other Brazilian epidemiological studies [15,16]. This pattern is explained by greater mobility and involvement in higher-risk activities, such as agriculture and construction work, which increase exposure among these individuals [15-17]. However, the incomplete field for work-related exposure in 217 forms limited the analysis of this association.

The survey indicated that the hands/fingers were the body region most affected by scorpion stings, whereas the feet/toes were the most affected sites in spider and snakebite cases. These findings are consistent with the behavior of these animals, as scorpions frequently shelter in debris and crevices, increasing the likelihood of stings when handling objects, whereas spiders and snakes tend to remain close to the ground, including inside footwear [18]. In peri-urban and rural areas, the habit of walking barefoot may have increased the risk of spider and snakebite accidents. Among snakes, species of the genera *Bothrops* and *Crotalus*, which have terrestrial and cryptic habits, were particularly notable, predisposing individuals to lower-limb injuries when the animals are stepped on [18]. This pattern is relevant to guiding educational interventions, such as promoting footwear use, conducting shoe inspections, and proper waste management.

The results showed that most victims sought treatment within three hours after the bite or sting, with a large proportion of cases classified as mild, regardless of the type of venomous animal involved. This finding is positive, as treatment effectiveness depends on timely access to healthcare and is essential for a favorable prognosis [19]. A concerning finding was the incompleteness of the “case severity” field: a substantial proportion of forms were blank, making it difficult to confirm whether most cases were truly mild, particularly in scorpion- and spider-related accidents. 

The lack of information on spider species was one of the main deficiencies identified, as most forms left this field blank or marked it as unknown, a pattern previously reported [17]. This finding reinforces the need for training in recognizing clinical manifestations associated with different animal types and, whenever possible, for direct identification of the animal or identification through photographs provided by patients. Among the identified cases, *Loxosceles* was the genus responsible for the most accidents, as previously reported [15,17]. The predominance of this genus has important clinical implications, as brown spider envenomation has been associated with potentially severe presentations, including systemic complications and death [17]. 

Among snakes, lancehead bites were the most frequent (40%), owing to the wide distribution and defensive behavior of the genus *Bothrops* [18]. Although lancehead snakes are responsible for most snakebite envenomations in Brazil, accounting for approximately 70% of cases [20], an increase in crotalic accidents has been observed in the inland areas of the State of Rio de Janeiro, rising from 9% to 40% of snakebite cases over seven years of investigation [21]. During the study period, the proportions of lancehead (40%) and rattlesnake (30%) cases were comparable. Rattlesnakes represented the species associated with the highest risk of death in the Southeast region [2] due to the neurotoxic effects of their venom. This finding highlights the need to maintain high availability of crotalic antivenom and to strengthen healthcare services capable of recognizing early signs of severe envenomation in these cases.

Higher rates of scorpion stings were observed at the end of the dry season and during warmer months [16,17]. However, no clear seasonal fluctuation was observed in the incidence of spider and snakebite cases, as may occur in regions with more pronounced climatic seasonality, such as the Amazon or semi-arid regions [15,22]. Temporal variation in cases was not uniform across the national territory; in areas with relatively stable land occupation and continuous human activity, the risk of accidents may be distributed throughout the year [15,17]. The low variability in the mean incidence of spider and snakebite cases and the year-round occurrence of these envenomations should be considered when planning antivenom availability across all seasons. 

Antivenom administration in mild cases of scorpion stings (n=18; 26.9%) and spider envenomations (n=4; 36.4%) was noteworthy. This was contrary to guidelines that recommend its use only for moderate and severe cases of these conditions [17]. This finding revealed an important discrepancy between technical recommendations and observed clinical practice in the studied service. Administering antivenom in cases without clinical indication exposes patients to significant risks, such as anaphylaxis and serum sickness, and negatively impacts both resource utilization and stock availability for cases in which it is truly needed [22]. 

Inappropriate antivenom use remains frequent in emergency care services, especially in contexts of diagnostic uncertainty or lack of familiarity with clinical guidelines [22,24]. This finding highlights the need for continuous investment in training healthcare teams, with emphasis on clinical management guidelines, severity classification, and appropriate indications for antivenom therapy.

The high incompleteness observed in notification forms, particularly in the fields related to systemic manifestations, local complications, systemic complications, educational level, race/color, occupation, and case outcome-classified as “poor” or “very poor”-represented another important finding of this study. Poor completion of fields related to social determinants, such as education, occupation, and race/color, hinders the formulation of public health policies to implement prevention strategies for the most vulnerable populations [24,25]. The lack of information on case outcomes and complications hampers the assessment of case severity and, consequently, may compromise the planning and appropriate distribution of antivenoms [24].

Regarding mandatory fields, the high proportion of blank entries on paper-based forms suggests weaknesses in the information-recording process. Considering that these fields are required for case notification and closure in the Sinan [4], the findings indicate the need for greater attention to the recording process and its supervision [26]. One hypothesis is that in clinical fields, the absence of signs and symptoms leads professionals to leave the fields blank rather than mark “no”.

Lack of understanding of the importance of proper reporting and insufficient supervision contributed to low data completeness [25,26]. Incomplete reporting also reflects management weaknesses, such as the absence of systematic monitoring mechanisms and a lack of clear assignment of responsibility for completing notification forms [24,27]. In this context, it is essential to train healthcare teams to improve both form completion and clinical management of cases. Continuing education and periodic audits may improve data quality, standardize recorded information, and strengthen adherence to surveillance routines [24-25,27]. Making completion of essential fields in the Sinan system mandatory would substantially reduce notification incompleteness and improve the reliability of generated indicators [24,25]. 

A limitation of this study was the use of data from the COVID-19 pandemic period, marked by social distancing and reduced use of nonessential health services [28], which may have led to underreporting of envenomation caused by venomous animals. However, while a stable trend in the incidence of these conditions was observed in the Southeast region during the pandemic [29], a reduction in scorpion stings and spider envenomations was reported nationwide [30], suggesting that the pandemic’s impact on reporting may have varied regionally. The data used in this study were obtained directly from paper-based notification forms, which had not yet been entered into the Sinan system and were completed by different professionals during patient care. Therefore, the study was subject to limitations inherent in the use of secondary data, such as potential inconsistencies and incomplete field completion. 

This study highlighted the predominance of scorpion stings and the relevance of this condition as a persistent public health problem in the Centro-Sul region of the State of Rio de Janeiro. Although most cases were classified as mild and received prompt care, antivenom was used in situations without clinical indication, revealing weaknesses in therapeutic management and adherence to Ministry of Health guidelines. In parallel, the high level of incompleteness in notification forms compromised the assessment of case severity and the planning of antivenom distribution. The lack of identification of the species involved further underscores the need to train healthcare professionals to improve the diagnosis and clinical management of envenomation cases. Therefore, improving reporting practices and clinical conduct through updated protocols, training, and regular supervision is essential to promote the rational use of resources, strengthen surveillance, and ensure safer, more efficient patient care.

## Data Availability

The database used in this study is available in the Figshare repository at the following link: https://doi.org/10.6084/m9.figshare.28655264 [12].
